# AST: Activity-Security-Trust driven modeling of time varying networks

**DOI:** 10.1038/srep21352

**Published:** 2016-02-18

**Authors:** Jian Wang, Jiake Xu, Yanheng Liu, Weiwen Deng

**Affiliations:** 1 College of Computer Science and Technology, Jilin University, Changchun 130012, China; 2 Key Laboratory of Symbolic Communication and Knowledge Engineering of Ministry of Education, Jilin University, Changchun 130012, China; 3 State Key Laboratory of Automotive Simulation and Control, Jilin University, Changchun 130012, China

## Abstract

Network modeling is a flexible mathematical structure that enables to identify statistical regularities and structural principles hidden in complex systems. The majority of recent driving forces in modeling complex networks are originated from activity, in which an activity potential of a time invariant function is introduced to identify agents’ interactions and to construct an activity-driven model. However, the new-emerging network evolutions are already deeply coupled with not only the explicit factors (e.g. activity) but also the implicit considerations (e.g. security and trust), so more intrinsic driving forces behind should be integrated into the modeling of time varying networks. The agents undoubtedly seek to build a time-dependent trade-off among activity, security, and trust in generating a new connection to another. Thus, we reasonably propose the Activity-Security-Trust (AST) driven model through synthetically considering the explicit and implicit driving forces (e.g. activity, security, and trust) underlying the decision process. AST-driven model facilitates to more accurately capture highly dynamical network behaviors and figure out the complex evolution process, allowing a profound understanding of the effects of security and trust in driving network evolution, and improving the biases induced by only involving activity representations in analyzing the dynamical processes.

Since the end of 20″ century, scientists have been committed to addressing the complex network modeling problems^1^,^2^,^3^,^4^,^5^,^6^,^7^,^8^,^9^. Erdős and Rényi^10^ regarded the network represented by a graph as a whole and proposed the concept of random network named Erdős-Rényi model through mathematics. In the following 40 years, the random-graph methodology occupies the foundation of graph theory. The random network that plays a great role in promoting the network modeling was advancing with a huge development, from Erdős-Rényi model, Logit models, p*-models, to Markov random graphs model^10^,^11^,^12^,^13^. Watts and Strogatz^14^ published the small-world network model in 1998 of Nature and Barabdsi and Albert^15^ proposed the scale-free network in 1999 of Science, which marks the birth of complex networks. From then on, the complex networks have switched to be the mainstream tool for the study of complex systems^16^. The scale-free network laid the foundation for the development of the connectivity-driven network that is quite fashionable in recent years. The connectivity-driven network mainly pays much attention to the topological structure that inspires the design and definition of various modeling algorithms, and thus those models facilitate to capture the essential features underlying stable systems e.g. the Internet, where the connections between nodes are of persistent partnership^17^,^18^,^19^. However, in many cases the interactions among elements are only active at certain points in time and are characterized by intermittent activation at the scale of individual links and short-lived duration^20^,^21^,^22^, such as time varying networks. Time varying networks are of particular relevance to propagation processes, e.g. the dissemination of information and disease, since each link is a contact opportunity and the time sequences of contacts are included. To mitigate this limitation, Perra^23^ proposed the activity-driven network model that involves the activity pattern of nodes and enables to explicitly model the evolution process of the connectivity over time. Moreover, Perra^23^ analyzed three large-scale, time-resolved network datasets and defined the concept of activity potential for each node to characterize the interaction pattern within the network. However again, in many new-emerging cases where active contacts are at risk, the interactions among nodes are not only dependent on the explicit factors (e.g. activity) but also restricted to the implicit considerations (e.g. security and trust). So the previous activity criterion is not only one key driving force in affecting the evolution process any more. The security of nodes and the mutual trust between each other become the indispensable push behind the screen to the generation and evolution of networks.

Creation of new links and strengthening of existing links in networks are important for the evolution of networks. The initial network is just a framework waiting for new elements to join in the collaboration, but relationships, learning, and sharing basic network quantities become rewards and incentives^24^. Theoretically, any network should at any time accelerate the interpersonal synergism through encouraging the creation of new connections and/or improving the existing links. Practically, this implies keeping up providing potential activity and/or initiating collaborations between little-previous-contact nodes. Normal evolution in networks would converge toward a scale-free structure, since some participants are natural hubs where some members themselves feel charming either by an attractive security level and/or by a favorable trust extent. The cautiousness resulted from security and trust considerations may pour cold water on the enthusiasm of eager connections if only caring about the activity. So we reasonably consider the activity, security, and trust of and between nodes comprehensively as three conjoint driving forces in network evolutions. Additionally, two drawbacks are potentially available in most the previous network models. At one hand, they devote to abstracting the physical elements into the virtual conceptual models by simplifying and/or ignoring the ubiquitous constraints underlying the actual systems, by which fruitful results are harvested but difficultly applicable to the practice. At the other hand, they impose too many assumptions to easily understand and popularize the network models, which fail to accommodate a suitable mapping set between the physical elements and the virtual concepts. Therefore, the network modeling should properly balance between generalization and applicability.

The essence of network evolution is to determine when, where, and how to create new links and update the existing links. To overcome the aforementioned drawbacks, we encode the security and trust of nodes rather than only depending on activity, and establish a one-to-one mapping set between the characteristics of real networks and the parameters of network models. To this end, we propose the Activity-Security-Trust (AST) driven model, in which the set of active nodes reflects those that probably join in the network, the activity rate quantifies the possibilities of nodes initiating the connection, the security level indicates the probability of nodes receiving the connections, and the trust extent emphasizes the opportunity of two nodes building the connections. The AST-driven model facilitates to objectively and accurately characterize the evolution process of the target network.

## Results

We focus on analyzing three large-scale and time-resolved network datasets. The first dataset is composed of border routers and the undirected connections indicate at least one packet has been exchanged between the corresponding endpoint routers. The second dataset represents the undirected links connecting two users of Wikipedia if one votes for or against another in admin elections. The third network is obtained by drawing an undirected edge between any two employees that send e-mails to each other in a mid-sized manufacturing company. These datasets represent different types of networks. We define two measurable quantities for each node, the activity potential and the security level, and also allocate to each ordered pair nodes a measurable quantity, the trust extent. We find that the system-level dynamics can be disclosed by the activity potential distribution function from which the appropriate interaction rate among nodes is possibly derived, by the security-level distribution function from which it is possible to deduce the ability of resisting malicious attacks, and by the computational trust extent from which the effect of mutual trust on network evolution could be reasoning. Considering the empirically measured activity potential distribution, the security-level distribution, and the computational trust extent, we propose a process model for the generation and evolution of time varying networks, named Activity-Security-Trust (AST) driven model. The AST model timely regulates the network structure and traces to the source of hubs due to the heterogeneous activity, the asymmetrical security, and the coupled trust of and among the network elements. To assess the validity of the AST model, we compare the topological characteristics of three real datasets and the AST model. The results show that the AST model is capable of objectively reflecting the evolution process of real networks.

### The activity potential

Perra^23^ presented the definition of activity potential and accordingly proposed the activity-driven network model. Similarly, we consider activity as an explicit driving force and follow the concept of activity potential in the AST model. The activity means the individual activity completing through various cooperation with others. Sufficient evidences for the role of activity in network modeling can be readily observed in the collaboration network of scientific authors^25^. We investigate three dataset networks in which the individual activity can be measured respectively, i.e., traffic flow exchanged among Autonomous Systems (AS) collected from University of Oregon Route Views Project - Online data and reports, voting actions for or against each other in admin elections of English Wikipedia, and e-mail delivery from one employee to another. For each dataset, we quantify the individual activity of each node and define the activity potential *x*_*i*_ of node *i* as the number of interactions *I*_*i*_(Δ*t*) that agent *i* performs in a characterized time window Δ*t*, divided by the total number of interactions *U*(Δ*t*) of all agents during the same time window Δ*t*. *x*_*i*_ is expressed by^23^:


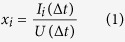


The activity potential *x*_*i*_ is an inherent property representing whether or not nodes are willing to collaborate with others, like human being’s introversion and extroversion. The value of *x*_*i*_ cannot happen to change upon node *i* birth. The larger the value of *x*_*i*_ is, the more actively the node connects to another. The probability distribution *F*(*x*) that a given element *i* has activity potential *x*_*i*_ statistically captures the interaction dynamics, as expressed by:


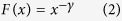


where *γ* is a factor, 1 < *γ* < 3, which is only dependent on the type of networks. *F*(*x*) may be formed arbitrarily or fitted by empirical data. We attach a lower cut-off *ε* on *x* in order to avoid possible divergence of *F*(*x*) at close to the origin, i.e. *ε* ≤ *x* ≤ 1. The term *a*_*i*_ indicates the activ_*i*_ty rate of node *i*, and is defined as the probability per unit time to create new links or interactions with others. The value of *a*_*i*_(*t*) is time-dependent and affected by *x*_*i*_, and should gradually climb up to a stable point as the degree of node *i* increases. So the definition of *a*_*i*_(*t*) is expressed by:


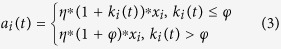


where *η* is a rescaling factor, *η* > 0, *k*_*i*_(*t*) is the degree of node *i* at time *t*, and *φ* restricts the allowable maximum value of *a*_*i*_(*t*).

### The security level

The security is a specialized field consisting of the provisions and policies to prevent unauthorized access, misuse, tamper, and denial of a computer network and network-accessible resources as well as ensuring their availability through proper procedures^26^. In the AST model, the security level emphasizes the ability against malicious elements. Like activity potential *x*_*i*_, the security level *y*_*i*_ is an intrinsic quantity of node *i*, and generally keeps frozen unless initiative to strengthen the security level by the node itself. Much literature^27^,^28^,^29^ about the quantification of security level in various networks are available, by which we can specify a security-level quantity *y*_*i*_ for each node, and formalize the security-level probability distribution function *L*(*y*) that deduces the ability of resisting malicious attacks, as expressed by:


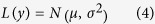


where *N* (*μ, σ*^*2*^) is a normal distribution with expectation *μ* and variance *σ*^*2*^. The values of *μ* and *σ* are determined by the served network type. Network connections introduce the possibility of cascading failures due to an exogenous or endogenous attack^30^, which implies the more active node is more prone to suffer from being attacked by malicious nodes due to possess numerous contacts. We define threat *z*_*i*_ represent the amount and intensity of the suffered attacks to node *i*. The value of threat *z*_*i*_ should gradually worsen to a saturation point as the degree of node *i* increases, as expressed by:


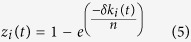


where 0 ≤ *z*_*i*_ ≤ 1, *n* indicates the number of potential nodes that may become active in the successive evolution, and *δ* is a factor, 10 ≤ *δ* ≤ 20, which is decided by the target network type. We employ the robustness *s*_*i*_(*t*) to quantify the possibility that node *i* is not infected by malicious nodes at time *t*, as expressed by:


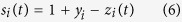


where 0 ≤ *s*_*i*_ ≤ 2. The stronger the security level of the node is, the more alleviated the threat is, the more improved the robustness is, and the less likely to be infected by malicious nodes.

### The trust extent

In social science, the trust is considered as an asymmetrical dependency relationship, and constitutes the cornerstone of network evolution, so we quantify the mutual trust extent between each other in the AST model. The extent to which one agent trusts another is a measure of belief in the honesty, fairness, or benevolence of another party. Trust is an elemental consideration in approving a connection construction. The trust extent emphasizes the opportunity of the two nodes building the connections. Essentially, the trust extent can be shaped by two means: through its own enough ability to win partners’ trust, and/or through the frequent contact with others. The contact frequency can be quantified by the times of two nodes interacting during a time interval. Therefore, the trust extent *b*_*ij*_(*t*) of node *i* on node *j* at time *t* is defined by:


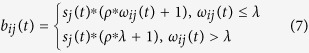


where *ρ* is a factor weighting the contributions between the number of connections and the security level to the trust extent. The bigger the value of *ρ* is, the more significant effect the connections exert on the trust extent. *λ* restricts the allowable maximum number of the related connections to the trust extent. *ω*_*ij*_(*t*) is the total number of connections between node *i* and node *j* before time *t*, as expressed by:


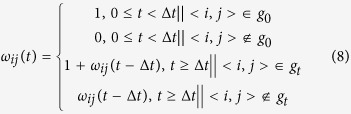


where *g*_0_ represents the initial network, <*i*, *j*> is a edge connecting node *i* and node *j*, *g*_*t*_ represents the instantaneous network at time *t*, and Δ*t* is the time span of generating the instantaneous network.

### Activity-Security-Trust driven network model

**Figure 1:**
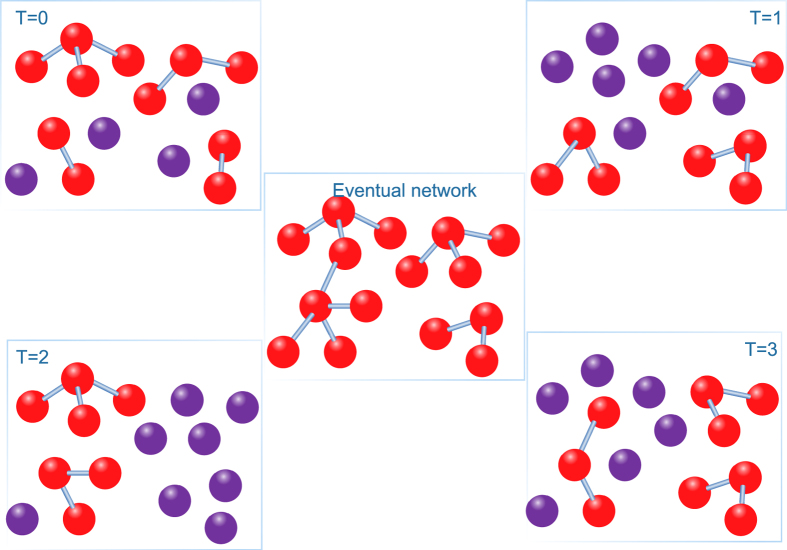
A schematic representation of the AST model. Considering 12 nodes and *m* = 2, we visualize the result of the initial network and other three different instantaneous snapshots, where the red nodes indicate the active nodes. The final visualization represents the eventual network over all time steps.

We show the dynamic network generation process (see Fig. 1).

Step i. Initialize the number of potential nodes, the activity probability distribution *F*(*x*), and the security-level probability distribution *L*(*y*).

Step ii. According to *F*(*x*), assign the activity potential *x*_*i*_ for each potential node *i*.

Step iii. According to *L*(*y*), assign the security level *y*_*i*_ for each potential node *i*.

Step iv. Regard the initial network *g*_0_ introduced from the actual network as the initial case of the eventual network *G*_*T*_.

Step v. Successively generate 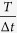 instantaneous network *g*_*t*_ (*t* = Δ*t*, 2Δ*t*, 3Δ*t*, …, *T*).

Step vi. Generate the eventual network 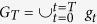.

where *T* is the time span of generating the eventual network, namely network aggregation time. Next we provide the creation process of an instantaneous network *g*_*t* + Δ*t*_ (*t* = 0, Δ*t*, 2Δ*t*, 3Δ*t*, …, *T *− Δ*t*).

Step i. At each discrete time step Δ*t*, the network *g*_*t* + Δ*t*_ starts with *n* disconnected vertices.

Step ii. Calculate degree *k*_*i*_(*t*) for each potential node and weight *ω*_*ij*_(*t*) for each edge in the eventual network *G*_*t*_.

Step iii. By *k*_*i*_(*t*), (5) and (6), calculate threat *z*_*i*_(*t*) and robustness *s*_*i*_(*t*) for each potential node *i*.

Step iv. By *ω*_*ij*_(*t*) and (7), calculate trust extent *b*_*ij*_(*t*) for each ordered pair of potential nodes.

Step v. By *k*_*i*_(*t*) and (3), calculate activity rate *a*_*i*_(*t*) for each potential node *i*.

Step vi. Determine the active node in the probability *a*_*i*_(*t*)Δ*t*, otherwise become the black-hole node in the probability 1-*a*_*i*_(*t*)Δ*t*, i.e. only passively wait for receiving connections from active nodes.

Step vii. Create *m* connections for each active node *i* in terms of the independent probability *Q*_*ij*_(*t*), and attach the corresponding edges to the instantaneous network *g*_*t* + Δ*t*_. The independent selection infers that duplicate target nodes are possibly available in *m* connections.

Step viii. At the next time step Δ*t*, all the edges in the network *g*_*t* + Δ*t*_ are erased, by which it holds that all interactions have a constant duration Δ*t*.

where *Q*_*ij*_(*t*) is defined by:


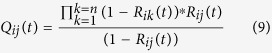


where *R*_*ij*_(*t*) is the trust-extent probability function, and expressed by:


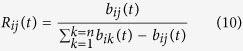


The trust-extent probability function *R*_*ij*_(*t*) indicates the proportion of node *i*’s trust extent on node *j* to the total trust, i.e. the more node *i* trusts node *j*, the bigger the value of *R*_*ij*_(*t*) is. One note is that the activity rate *a*_*i*_(*t*) may exceed over 1 after certain time *t*, and thus node *i* becomes dominantly active and is always hit in each selection, such as the hotspot servers in the Internet and the convergence routers in AS, which constantly connect to others.

The AST model outputs various random networks that share the same control parameters, however, the resulted eventual networks look different. Such differences are so small as to be statistically ignored in a large-scale complex network. The essence of network evolution is the process of generating new edges, which can be simplified into two sub-processes. One is to select an active node from potential nodes as the starting node of an edge, and the other is to select a terminal node from the rest. Accordingly, the activity rate affects the selection of the active node, while the security level and trust extent govern the determination of the target node. The AST model imitates the real generation process, and the parameters originate from actual networks, so the AST model is capable of objectively and accurately characterizing practical time varying networks.

**Figure 2:**
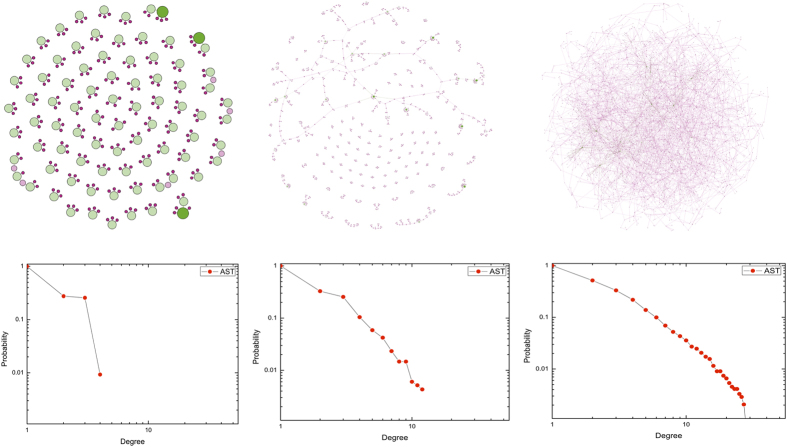
Visualization of the proposed AST model and the cumulative degree probability distribution against aggregation time *T*. In the top half, we show the network visualization of the AST model, and in the bottom half, we plot the corresponding cumulative degree probability distribution. We employ *g*_0_ = *Φ*, *n* = 4000, *m* = 3, *η* = 8, *γ* = 2.8, *φ* = 5, *μ* = 0.5, *σ* = 1, *δ* = 15, Δ*t* = 1, *ρ* = 20, *λ* = 10, and *ε* = 10^−3^. We respectively plot the network obtained after one time step (*T* = 1) in the left column, after integrating over 5 iterations (*T* = 5) in the middle, and after integrating over 10 iterations (*T* = 10) in the right.

Figure 2 provides the results of numerical simulations of the network against various aggregation time *T*, and Table 1 shows the corresponding statistical information. The cumulative degree probability distribution gradually becomes slowly as the aggregation time *T* increases, which implies that the network accelerates growing as the size is enlarged. The increased aggregation time positively affects the network centralization and network heterogeneity but negatively restricting the network density. At each time step, the network appears to a simple random graph with low average connectivity. The accumulation of connections during the long aggregation time *T* improves the activity rate of nodes, and worsens the security level reversely. Due to the heterogeneous activity and asymmetrical security of nodes, the hubs that possess a large activity rate and trust extent are born in the network.

The AST model supports simple analytical evaluation. We define the eventual network 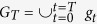 as the union of all the instantaneous networks generated during each previous time step Δ*t*. Then, we erase the duplicated edges and self-links. The instantaneous network is composed of a set of newly interconnected nodes that correspond to exactly being active at that time, plus those who received connections from active agents. Assuming *g*_0_ = *Φ* (i.e. an empty network without nodes or edges), each active node creates *m* (or less *m*) links and the total edges per unit time are *E*(*t*) ≈*m* < *a*(*t*)>, yielding the average degree per unit time 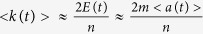. Here <*a*(t)> is the average activity rate per unit time.

## Discussion

The AST model is concise and understandable but not easy to determine the proper parameters so as to accurately reflect the real network characteristics. Fortunately, the AST parameters can be empirically measured in real world networks. One feasible way is to learn the driving forces governing the network evolution and then to symbolize the corresponding quantitative representation from priori knowledge. Another possible avenue to parameterization is to initially separate the evolution of existing networks into several short time durations, and then to determine network characteristics and parameters through constantly fitting parameters against the actual networks. Moreover, the AST model can be extensively used to research the molecular networks, time-varying networks and spatiotemporal network, and also facilities to predict epidemics dissemination and to investigate the human dynamics of face-to-face interaction networks. In summary, accurately understanding the network-evolution essence requires considering not only the explicit factors (e.g. activity potential) but also the implicit factors (e.g. security level and trust extent). The explicit factors reflect nodes’ subjective initiative to create connections with others, while the implicit factors emphasize nodes’ objective prudent to resolve the candidate targets. More factors are permitted to be associated with each connection decision (e.g. concurrency and persistence) in order to melt the limitations underlying the simple random networks. But one note is that the network modeling should properly balance between generalization and applicability, which represents interesting challenges for future work in this area.

## Methods

### Datasets

We compare the AST model with three datasets (Supplementary Information): traffic flow exchanged among ASs collected from University of Oregon Route Views Project - Online data and reports, voting for and against each other in admin elections of English Wikipedia, and E-mails of employees in a mid-sized manufacturing companies to each other. We mainly focus on the number of nodes and the corresponding degree distribution in the undirected and unweighted graph, so we employ the cumulative degree distribution as a measure of topological similarity, in which the number of the nodes with one degree is exactly equal to the total number of nodes. For a given dataset, only the potential nodes *n* and the factor *η* could happen to change in adjusting the aggregation time, but not the other parameters due to their being the inherent properties of networks. According to the empirically measured network-specific properties, we give the parameters of the three datasets. The parameters of the ASs dataset are *m* = 1, *γ* = 1.7*, φ* = 100, *μ* = 0.5, *σ* = 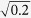, *δ* = 20, Δ*t* = 2, *ρ* = 10, *λ* = 2000, and *ε* = 10^−3^. The parameters of Wikipedia elections dataset are *m* = 3, *γ* = 2.7, *φ* = 50, *μ* = 0.5, *σ* = 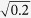, *δ* = 15, Δ*t* = 1, *ρ* = 150, *λ* = 100, and *ε* = 10^−3^. The parameters of manufacturing company E-mails dataset are *m* = 4, *γ* = 1.2, *φ* = 5, *μ* = 0.5, *σ* = 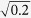, *δ* = 15, Δ*t* = 10, *ρ* = 50, *λ* = 30, and *ε* = 10^−3^.

### Autonomous systems dataset (AS)

**Figure 3:**
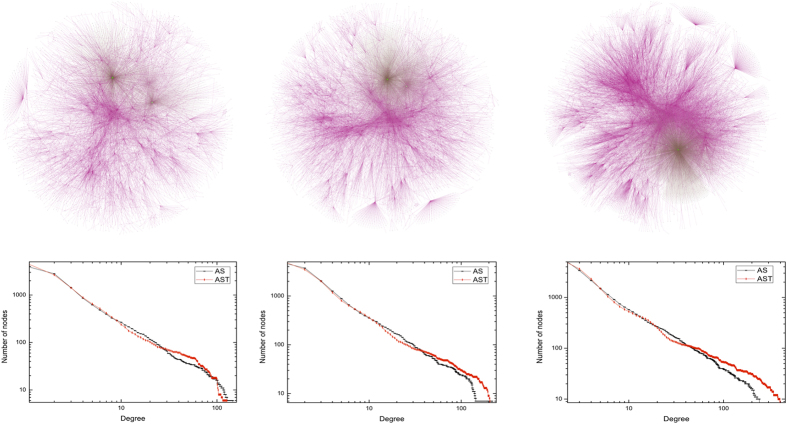
Network visualization and the cumulative degree distribution of AS dataset as well as AST model against three different aggregated views. In the top half, we show the network visualization of the AS dataset from the view of three different aggregated time durations. In the bottom half, we plot the cumulative degree distribution of the AS dataset as well as the AST model network. The left column corresponds to the network over 201 days, from November 8, 1997 to June 1, 1998, where *n* = 7750, and *η* = 0.0275. The middle column shows the network over 401 days, from November 8, 1997 to June 24, December 1998, where *n* = 7750, and *η* = 0.017. The right column indicates the network over 733 days, from November 8, 1997 to January 2, 2000, where *n* = 7750, and *η* = 0.012.

This dataset^31^ is composed of border routers and the undirected connections indicate at least one packet has been exchanged between the corresponding endpoint routers. The dataset contains 733 daily instances spanning 785 days from November 8, 1997 to January 2, 2000. We focus on three periods between 1997 and 2000. Table 2 shows the metadata of three periods. Figure 3 shows the network visualization and the cumulative degree distribution of the AS dataset as well as the AST model against three different aggregated views.

### Wikipedia elections dataset (Wiki)

**Figure 4:**
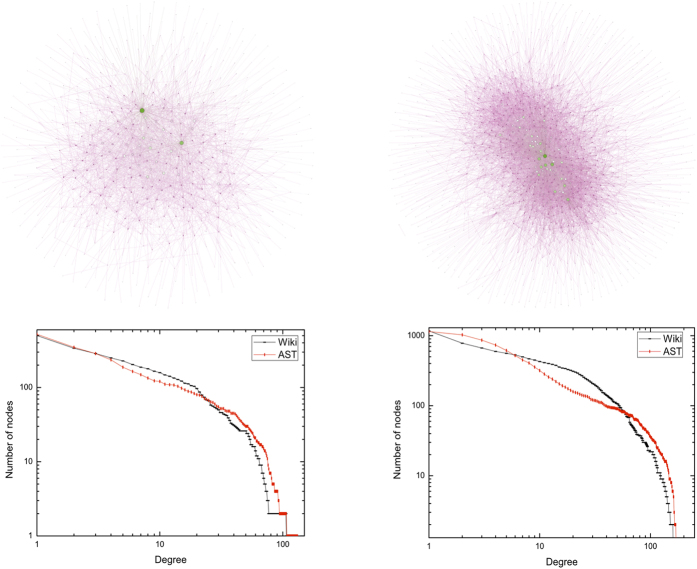
Network visualization of Wikipedia elections dataset and AST model against two different aggregated views. In the top half, we show the network visualization of Wiki dataset from the view of two different aggregated time durations. In the bottom half, we plot the cumulative degree distribution of the Wiki dataset and the AST network. The left column corresponds to the network over 92 days, from March 1, 2005 to May 31, 2005, where *n* = 750, and *η* = 1.7. The right column shows the network over 214 days, from March 1, 2005 to September 30, 2005, where *n* = 1200, and *η* = 1.

The Wiki dataset^32^ represents the undirected links connecting two users of Wikipedia if one votes for or against another in admin elections. Edges can be positive (“for” vote) and negative (“against” vote), but we treat both as the same. We consider two periods from March 1, 2005 to September 30, 2005. Table 3 shows the metadata of two periods. Figure 4 shows the network visualization of Wiki dataset and AST model against two different aggregated views.

### Manufacturing company E-mail dataset (E-mail)

**Figure 5:**
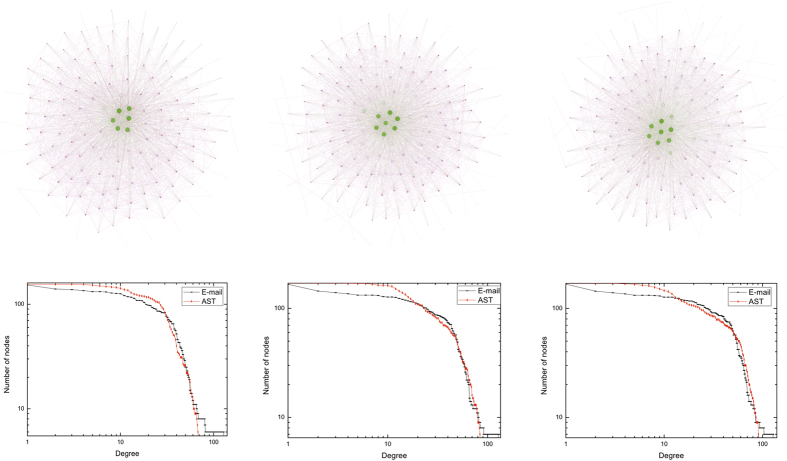
Network visualization of manufacturing company E-mail dataset and AST model against three different aggregated views. In the top half, we show the network visualization of E-mail dataset from the view of three different aggregated time durations. In the bottom half, we plot the cumulative degree distribution of the E-mail dataset and the AST network. The left column corresponds to the network over 90 days, from January 1, 2010 to March 31, 2010, where *n* = 155, and *η* = 1.3. The middle column shows the network over 181 days, from January 1, 2010 to June 30, 2010, where *n* = 170, and *η* = 0.54. The right column indicates the network over 273 days, from January 1, 2010 to September 30, 2010, where *n* = 170, and *η* = 0.3.

This dataset^33^ considers each employee of a mid-sized manufacturing company as a node. An undirected link exists if two employees sent e-mail to each other. We focus on three periods covering nine full months span from January 1, 2010 to September 30, 2010. Table 4 shows the metadata of three periods. Figure 5 shows network visualization of the E-mail dataset and AST model against three different aggregated views.

## Additional Information

**How to cite this article**: Wang, J. *et al.* AST: Activity-Security-Trust driven modeling of time varying networks. *Sci. Rep.* **6**, 21352; doi: 10.1038/srep21352 (2016).

## Supplementary Material

Supplementary Information

## Figures and Tables

**Table 1:** The statistical information of AST model.

Network aggregation time	Network centralization	Network density	Connected components	Network heterogeneity
1	0.008	0.005	74	0.580
5	0.009	0.002	93	0.874
10	0.011	0.001	3	1.142

**Table 2:** The metadata of AS dataset.

Nodes	Edges	Begin	End	Duration (day)
4094	9284	1997.11.8	1998.6.1	201
5143	12833	1997.11.8	1998.12.24	401
7716	21466	1997.11.8	2000.1.2	733

**Table 3:** The metadata of Wikipedia elections dataset.

Nodes	Edges	Begin	End	During (days)
501	2698	2005.3.1	2005.5.31	92
1168	9082	2005.3.1	2005.9.30	214

**Table 4:** The metadata of manufacturing company E-mail dataset.

Nodes	Edges	Begin	End	Duration (days)
153	2503	2010.1.1	2010.3.31	90
166	2989	2010.1.1	2010.6.30	181
167	3271	2010.1.1	2010.9.30	273

## References

[b1] Newman M. E. J. Networks: an introduction. (Oxford University Press, 2010).

[b2] Donner R. V. *et al.* Recurrence-based time series analysis by means of complex network methods. Int. J. Bifurcat. Chaos 21, 1019–1046 (2011).

[b3] Pagani G. A. & Aiello M. The power grid as a complex network: a survey. *arxiv*:1105.3338 (2011).

[b4] Yazdani A. & Jeffrey P. Complex network analysis of water distribution systems. Chaos 21, 016111 (2011). 21456853 10.1063/1.3540339

[b5] Gao Z. K. & Jin N. D. A directed weighted complex network for characterizing chaotic dynamics from time series. *Nonlinear Anal*. Real 13, 947–952 (2012).

[b6] Vespignani A. Modelling dynamical processes in complex socio-technical systems. Nat. Phys. 8, 32–39 (2012).

[b7] Boccaletti S., Latora V., Moreno Y., Chavez M. & Hwang D.-U. Complex networks: Structure and dynamics. Phys. Rep. 424, 175–308 (2006).

[b8] Liu Y. Y., Slotine J. J. & Barabási A. L. Controllability of complex networks. Nature 473, 167–173 (2011). 21562557 10.1038/nature10011

[b9] Pastor-Satorras R., Castellano C., Mieghem P. V. & Vespignani A. Epidemic processes in complex networks. Rev. Mod. Phys. 87, 925 (2015).

[b10] Erdős P. & Rényi A. On the evolution of random graphs. Selected Papers of Alfréd Rényi 2, 482–525 (1976).

[b11] Molloy M. & Reed B. A critical point for random graphs with a given degree sequence. Random Struct. Algor. 6, 161–180 (1995).

[b12] Holland P. W. & Leinhardt S. An exponential family of probability distributions for directed graphs. J. Am. Stat. Assoc. 76, 33–50 (1981).

[b13] Frank O. & Strauss D. Markov graphs. J. Am. Stat. Assoc. 81, 832–842 (1986).

[b14] Watts D. J. & Strogatz S. H. Collective dynamics of ‘small-world’networks. Nature 393, 440–442 (1998). 9623998 10.1038/30918

[b15] Barabási A. L. & Albert R. Emergence of scaling in random networks. Science 286, 509–512 (1999). 10521342 10.1126/science.286.5439.509

[b16] Pereira T., Eroglu D., Bagci G. B., Tirnakli U. & Jensen H. J. Connectivity-driven coherence in complex networks. Phys. Rev. Lett. 110, 234103 (2013). 25167497 10.1103/PhysRevLett.110.234103

[b17] Albert R., Jeong H. & Barabási A. L. Internet: Diameter of the world-wide web. Nature 401, 130–131 (1999).

[b18] Abbagnale A. & Cuomo F. Connectivity-driven routing for cognitive radio ad-hoc networks. In SECON 2010, Boston, MA. 10.1109/SECON.2010.5508269. (2010, June).

[b19] Holme P., Edling C. R. & Liljeros F. Structure and time evolution of an Internet dating community. Social Networks 26, 155–174 (2004).

[b20] Pan R. K. & Saramäki J. Path lengths, correlations, and centrality in temporal networks. Phys. Rev. E 84, 016105 (2011). 10.1103/PhysRevE.84.016105 21867255

[b21] Colizza V. & Vespignani A. Invasion threshold in heterogeneous metapopulation networks. Phys. Rev. Lett. 99, 148701 (2007). 17930732 10.1103/PhysRevLett.99.148701

[b22] Holme P. & Saramäki J. Temporal networks. Phys. Rep. 519, 97–125 (2013).

[b23] Perra N., Gonçalves B., Pastor-Satorras R. & Vespignani A. Activity driven modeling of time varying networks. Sci. Rep. 2, 469 (2012). 22741058 10.1038/srep00469 PMC3384079

[b24] Haemmerli B., Raaum M. & Franceschetti G. Effective surveillance for homeland security: balancing technology and social issues (eds. Flammini F. *et al.*) Ch. 2, 21-50 (CRC Press, 2013).

[b25] Newman M. E. J. The structure of scientific collaboration networks. Proc. Natl. Acad. Sci. USA 98, 404–409 (2001). 11149952 10.1073/pnas.021544898 PMC14598

[b26] Kumar G. & Kumar K. Network security–an updated perspective. Syst. Sci. Contr. Eng. 2, 325–334 (2014).

[b27] Madan B. B., Gogeva-Popstojanova K., Vaidyanathan K. & Trivedi K. S. Modeling and quantification of security attributes of software systems. In DSN 2002, Bethesda, MD. 10.1109/DSN.2002.1028941. (2002, June).

[b28] Madan B. B. & Trivedi K. S. Security modeling and quantification of intrusion tolerant systems using attack-response graph. J. High Speed Netw. 13, 297–308 (2004).

[b29] Verendel V. Quantified security is a weak hypothesis: a critical survey of results and assumptions. In NSPW 2009, Oxford, United Kingdom. 10.1145/1719030.1719036. (2009, September).

[b30] Acemoglu D., Malekian A. & Ozdaglar A. Network security and contagion. *National Bureau of Economic Research*, NBER Working Paper No. 19174. 10.3386/w19174. (2013, June).

[b31] Leskovec J., Kleinberg J. & Faloutsos C. Graphs over time: densification laws, shrinking diameters and possible explanations. In KDD 2005, Chicago, IL. 10.1145/1081870.1081893. (2005, August).

[b32] Leskovec J., Huttenlocher D. & Kleinberg J. Predicting positive and negative links in online social networks. In WWW 2010, Raleigh, NC. 10.1145/1772690.1772756. (2010, April)

[b33] Michalski R., Palus S. & Kazienko P. Matching organizational structure and social network extracted from email communication (ed. Abramowicz W.) 197–206 (Springer Berlin Heidelberg, 2011).

