# Supplementary material for: AST: Activity-Security-Trust driven modeling of time varying networks
Source: Sci Rep. 2016 Feb 18;6:21352. doi: 10.1038/srep21352 (PMC4758040; doi:10.1038/srep21352)
Supplement: Supplementary Information [file srep21352-s1.doc]

**Supplementary Information**

**AST: Activity-S****ecurity-Trust driven modeling of time varying networks**

Jian Wang, Jiake Xu, Yanheng Liu, Weiwen Deng

**1 The Model**

In this paper, we propose the Activity-Security-Trust (AST) driven model through synthetically considering the explicit and implicit driving forces (e.g. activity, security, and trust) underlying the decision process. AST is composed of one-to-one mapping set between the characteristics of the real network and the parameters of the network model to quantificationally characterize the entire evolution process. Concretely speaking, the set of active nodes reflects the potential nodes that probably join in the network, the activity rate quantifies the possibilities of nodes initiating the connection, the security level indicates the probability of nodes receiving the connections, and the trust extent emphasizes the opportunity of the two nodes building the connections.

We define two measurable quantities for each node, the activity potential and the security level, and also allocate to each ordered pair nodes a measurable quantity, the trust extent. We find that the system-level dynamics can be disclosed by the activity potential distribution function from which the appropriate interaction rate among nodes is possibly derived, by the security-level distribution function from which it is possible to deduce the ability of resisting malicious attacks, and by the computational trust extent from which the effect of mutual trust on network evolution could be reasoning. The AST model timely regulates the network structure and traces to the source of hubs due to the heterogeneous activity, the asymmetrical security, and the coupled trust of and among the network elements.

We show the dynamic network generation process.

Step i. Initialize the number of potential nodes, the activity probability distribution *F*(*x*), and the security-level probability distribution *L*(*y*).

Step ii. According to *F*(*x*), assign the activity potential *x_i_* for each potential node *i*.

Step iii. According to *L*(*y*), assign the security level *y_i_* for each potential node *i*.

Step iv. Regard the initial network *g*_0_ introduced from the actual network as the initial case of the final eventual network *G_T_*.

Step v. Successively generate instantaneous network *g_t_* (*t*=Δ*t*, 2Δ*t*, 3Δ*t*, …, *T*).

Step vi. Generate the eventual network .

where *T* is the time span of generating the eventual network, namely network aggregation time. Next we provide the creation process of an instantaneous network *g_t_*_+Δ_*_t_* (*t*=0, Δ*t*, 2Δ*t*, 3Δ*t*, …, *T*-Δ*t*).

Step i. At each discrete time step Δ*t*, the network *g_t_*_+Δ_*_t_* starts with *n* disconnected vertices.

Step ii. Calculate degree *k_i_*(*t*) for each potential node and weight *ω_ij_*(*t*) for each edge in the eventual network *G_t_*.

Step iii. By *k_i_*(*t*), calculate threat *z_i_*(*t*) and robustness *s_i_*(*t*) for each potential node *i*.

Step iv. By *ω_ij_*(*t*), calculate trust extent *b_ij_*(*t*) for each ordered pair of potential nodes.

Step v. By *k_i_*(*t*), calculate activity rate *a_i_*(*t*) for each potential node *i*.

Step vi. Determine the active node in the probability *a_i_*(*t*)Δ*t*, otherwise become the black-hole node in the probability 1-*a_i_*(*t*)Δ*t*, i.e. only passively wait for receiving connections from active nodes.

Step vii. Create *m* connections for each active node *i* in terms of the independent probability *Q_ij_*(*t*), and attach the corresponding edge to the instantaneous network *g_t_*_+Δ_*_t_*. The independent selection infers that duplicate target nodes are possibly available in *m* connections.

Step viii. At the next time step Δ*t*, all the edges in the network *g_t_*_+Δ_*_t_* are erased, by which it holds that all interactions have a constant duration Δ*t*.

**2 Dataset Details**

We compare the AST model with three datasets: traffic flow exchanged among ASs collected from University of Oregon Route Views Project - Online data and reports, voting for and against each other in admin elections of English Wikipedia, and E-mails between employees in a mid-sized manufacturing company. We mainly focus on the number of nodes and the corresponding degree distribution in the undirected and unweighted graph, so we employ the cumulative degree distribution as a measure of topological similarity, in which the number of the nodes with one degree is exactly equal to the total number of nodes. For a given dataset, only the potential nodes *n* and the factor *η* could happen to change in adjusting the aggregation time, but not the other parameters due to their being the inherent properties of networks. According to the empirically measured network-specific properties, we give the parameters of the three datasets. The parameters of the ASs dataset are *m*=1, *γ*=1.7*, φ*=100, *μ*=0.5, *σ*=, *δ*=20, Δ*t*=2, *ρ*=10, *λ*=2000, and *ε*=10^-3^. The parameters of Wikipedia elections dataset are *m*=3, *γ*=2.7, *φ*=50, *μ*=0.5, *σ*=, *δ*=15, Δ*t*=1, *ρ*=150, *λ*=100, and *ε*=10^-3^. The parameters of manufacturing company E-mails dataset are *m*=4, *γ*=1.2, *φ*=5, *μ*=0.5, *σ*=, *δ*=15, Δ*t*=10, *ρ*=50, *λ*=30, and *ε*=10^-3^.

**2.1** **Autonomous systems dataset (AS)**

This dataset^1^ is composed of border routers and the undirected connections indicate at least one packet has been exchanged between the corresponding endpoint routers. The dataset contains 733 daily instances spanning 785 days from November 8, 1997 to January 2, 2000. We focus on three periods between 1997 and 2000. Table 1 shows the metadata of three periods. Fig. 1 shows the network visualization and the cumulative degree distribution of the AS dataset as well as the AST model against three different aggregated views.

**Table 1: The metadata of AS dataset**

| Nodes | Edges | Begin | End | Duration (day) |
| --- | --- | --- | --- | --- |
| 4094 | 9284 | 1997.11.8 | 1998.6.1 | 201 |
| 5143 | 12833 | 1997.11.8 | 1998.12.24 | 401 |
| 7716 | 21466 | 1997.11.8 | 2000.1.2 | 733 |

**
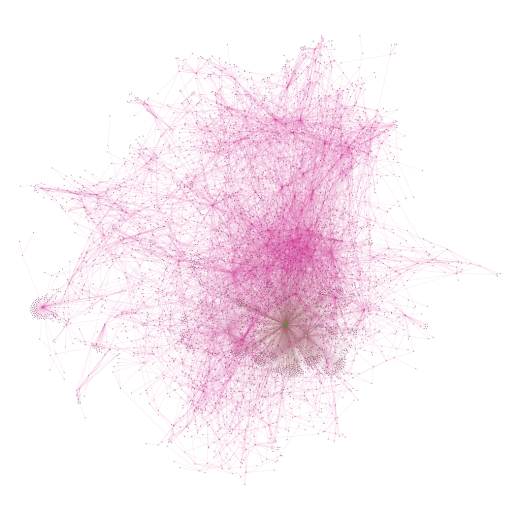

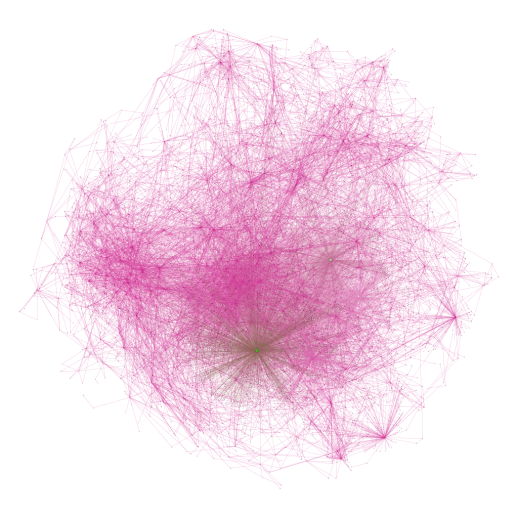

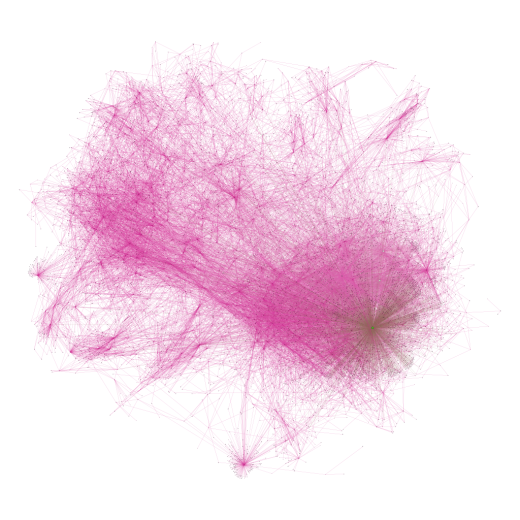
**

**Figure 1: Network visualization of AS dataset against three different aggregated views.** The left column corresponds to the network over 201 days, from November 8, 1997 to June 1, 1998, where *n*=7750, and *η*=0.0275. The middle column shows the network over 401 days, from November 8, 1997 to June 24, December 1998, where *n*=7750, and *η*=0.017. The right column indicates the network over 733 days, from November 8, 1997 to [January](javascript:void(0);) 2, 2000, where *n*=7750, and *η*=0.012.

**2.2 Wikipedia elections dataset (Wiki)**

The Wiki dataset^2^ represents the undirected links connecting two users of Wikipedia if one votes for or against another in admin elections. Edges can be positive ("for" vote) and negative ("against" vote), but we treat both as the same. We consider two periods from March 1, 2005 to [September](javascript:void(0);) 30, 2005. Table 2 shows the metadata of two periods. Fig. 2 shows the network visualization of Wiki dataset and AST model against two different aggregated views.

**Table 2: The metadata of** **Wiki** dataset

| Nodes | Edges | Begin | End | Duration (day) |
| --- | --- | --- | --- | --- |
| 501 | 2698 | 2005.3.1 | 2005.5.31 | 92 |
| 1168 | 9082 | 2005.3.1 | 2005.9.30 | 214 |

**
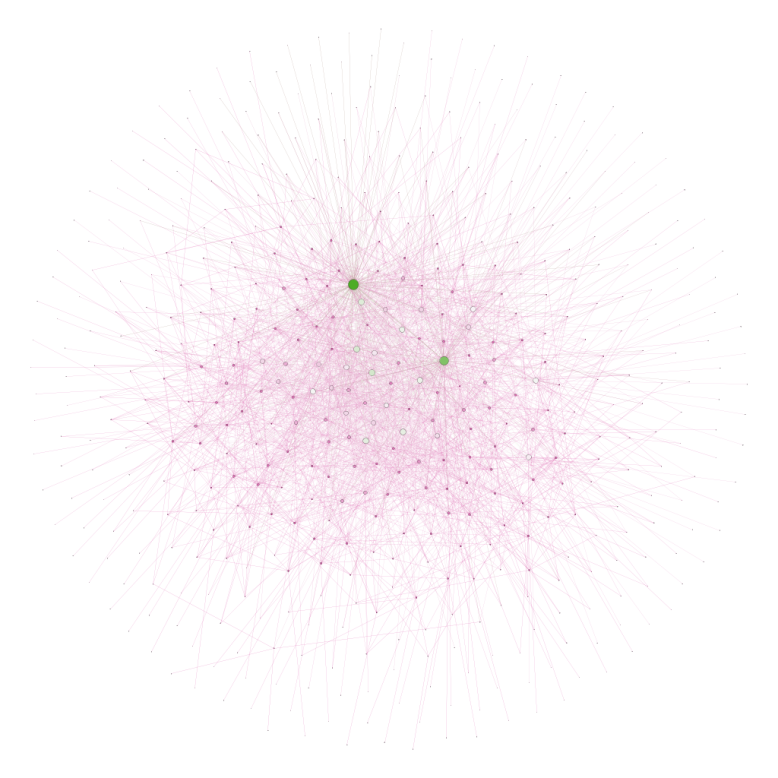

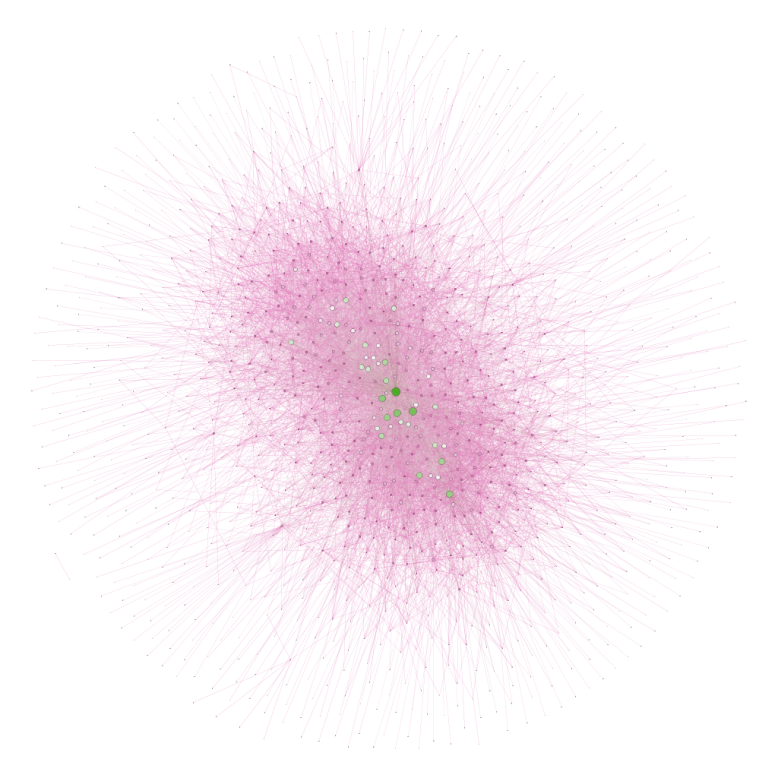
**

**Figure 2: Network visualization of Wiki dataset against two different aggregated views.** The left column corresponds to the network over 92 days, from March 1, 2005 to May 31, 2005, where *n*=750, and *η*=1.7. The right column shows the network over 214 days, from March 1, 2005 to [September](javascript:void(0);) 30, 2005, where *n*=1200, and *η*=1.

**2.3 Manufacturing company e-mail dataset (E-mail)**

This dataset^3^ considers each employee of a mid-sized manufacturing company as a node. An undirected link exists if two employees sent e-mail to each other. We focus three periods covering nine full months span from January 1, 2010 to September 30, 2010. Table 3 shows the metadata of three periods. Fig. 3 shows network visualization of the E-mail dataset and AST model against three different aggregated views.

**Table 3: The metadata of E-mail dataset**

| Nodes | Edges | Begin | End | Duration (day) |
| --- | --- | --- | --- | --- |
| 153 | 2503 | 2010.1.1 | 2010.3.31 | 90 |
| 166 | 2989 | 2010.1.1 | 2010.6.30 | 181 |
| 167 | 3271 | 2010.1.1 | 2010.9.30 | 273 |

**
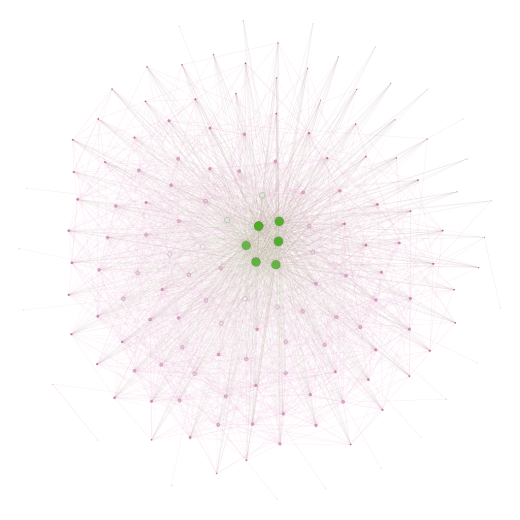

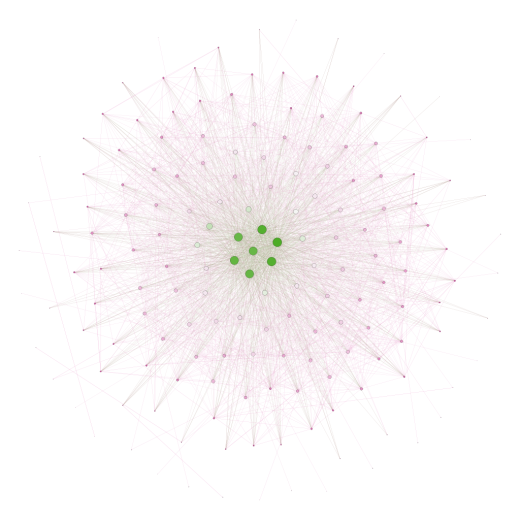

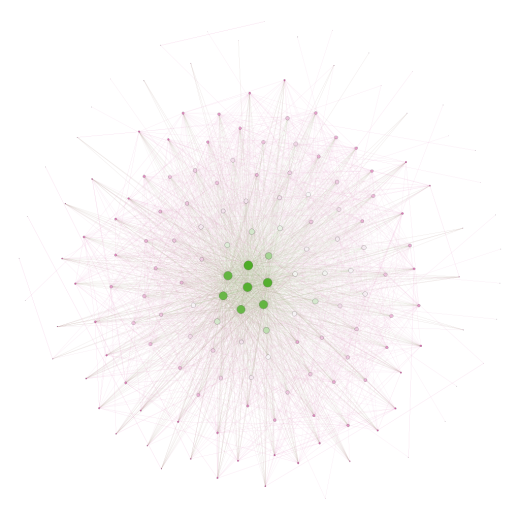
**

**Figure 3: Network visualization of E-mail dataset against three different aggregated views.** The left column corresponds to the network over 90 days, from January 1, 2010 to March 31, 2010, where *n*=155, and *η*=1.3. The middle column shows the network over 181 days, from January 1, 2010 to June 30, 2010, where *n*=170, and *η*=0.54. The right column indicates the network over 273 days, from January 1, 2010 to September 30, 2010, where *n*=170, and *η*=0.3.

References

[1] Leskovec, J., Kleinberg, J. & Faloutsos, C. Graphs over time: densification laws, shrinking diameters and possible explanations. In: *KDD 2005,* *Chicago, IL*. DOI: 10.1145/1081870.1081893. (2005, August)

[2] Leskovec, J., Huttenlocher, D. & Kleinberg, J. Predicting positive and negative links in online social networks. In: *WWW 2010, Raleigh, NC*. DOI: 10.1145/1772690.1772756. (2010, April)

[3] Michalski, R., Palus, S. & Kazienko, P. *Matching organizational structure and social network extracted from email communication* (ed. Abramowicz, W.) 197-206 (Springer Berlin Heidelberg, 2011).
